# One‐Step Tunable MoS_2_ with Enhanced Zn^2+^ Diffusion for High‐Energy Zinc‐Ion Hybrid Capacitors

**DOI:** 10.1002/advs.202506467

**Published:** 2025-09-23

**Authors:** Harshitha B. Tyagaraj, Vikram Mahamiya, Supriya J. Marje, Gagankumar Sakleshpur Kumar, Shalmali R. Burse, Swapnil R Patil, Ebrahim Al Hajri, Nilesh R. Chodankar, Yun Suk Huh, Young Kyu Han

**Affiliations:** ^1^ Department of Energy and Materials Engineering Dongguk University‐Seoul Seoul 04620 Republic of Korea; ^2^ Condensed Matter and Statistical Physics Section The Abdus Salam International Centre for Theoretical Physics (ICTP) Strada Costiera 11 Trieste 34151 Italy; ^3^ Mechanical and Nuclear Engineering Department Khalifa University of Science and Technology Abu Dhabi 127788 UAE; ^4^ Department of Biological Sciences and Bioengineering Nanobio High‐Tech Materials Research Center Inha University Incheon 22212 South Korea

**Keywords:** ethylene glycol intercalation, high energy density, layer spacing expansion, molybdenum disulfide, zinc‐ion hybrid capacitors

## Abstract

Zinc‐ion hybrid capacitors (ZIHCs) offer a promising solution for large‐scale energy storage, combining battery‐like energy density with superior power performance. However, their development is challenged by the scarcity of suitable cathode materials, as well as poor reversibility and sluggish Zn^2+^ diffusion due to its large hydrated ion size and limited efficiency. To address this, a one‐step, tunable synthesis approach is developed for growing MoS_2_ on carbon cloth via an ethylene glycol (EG) intercalation strategy, effectively transforming inactive interlayer cavities into highly active sites. Density functional theory calculations reveal that EG intercalation substantially lowers the energy barrier for hydrated Zn^2+^ intercalation, significantly improving Zn^2+^ storage capability. Experimentally, EG molecules expand the MoS_2_ interlayer spacing from 0.617 to 0.948 nm, creating wider diffusion channels for Zn^2+^ transport. The optimized EG‐MoS_2_ exhibits a high specific capacitance of 240.5 F/g at 0.7 A/g, which is three orders of magnitude higher than that of pristine MoS_2_, along with exceptional rate capability. Notably, the assembled ZIHC exhibited a high energy density of 40.42 Wh kg^−1^ at a power density of 385 W kg^−1^ while demonstrating outstanding cycling stability over 5000 cycles. This work unveils a powerful strategy for engineering high‐performance MoS_2_‐based cathodes, advancing next‐generation ZIHCs development.

## Graphical abstract

A one‐step, scalable method introduces ethylene glycol into MoS_2_ layers, expanding the interlayer distance from 0.617 to 0.948 nm. This modification enhances the diffusion of hydrated Zn^2+^ ions, significantly improving charge‐discharge kinetics.

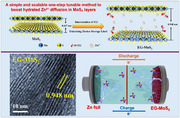

## 1 Introduction

To meet the demand for high‐speed development of clean, green, and sustainable energy technologies, the scientific community advocates for exploring advanced and cost‐effective electrochemical energy storage systems. These technologies hold significant potential across distinct applications, including wearable electronics, smart grid energy storage, and electric vehicles.^[^ ^1^, ^2^, ^3^ ^]^ In recent years, supercapacitors (SCs) and lithium‐ion batteries (LIBs) have emerged as the most promising electrochemical energy storage devices, owing to their excellent power density (≈10 W kg^−1^) and energy density (150–250 Wh kg^−1^), respectively. However, the limited energy density of SCs restricts their commercial application, while the short cycle life, high cost, and low reserve of lithium sources challenge the scalability of LIBs.^[^ ^4^, ^5^, ^6^ ^]^ Thus, LIBs and SCs do not simultaneously fulfill the requirements of high power density and long cycle life. To address these limitations, new energy storage technologies have been actively exploited.^[^ ^7^, ^8^ ^]^

Metal‐ion hybrid capacitors, which integrate the advantages of high energy density from battery‐type anodes and high‐power density from capacitor‐type cathodes, have progressively gained attention for clean energy storage. These systems utilize cations, including monovalent (e.g., Li^+^, Na^+^, and K^+^) and Multivalent (e.g., Mg^2+^, Ni^2+^, Zn^2+^, Ca^2+^, and Al^3+^) ions.^[^ ^9^, ^10^, ^11^, ^12^ ^]^ Among them, Zinc ion hybrid capacitors (ZIHCs), inspired by this hybrid energy storage mechanism, have been regarded as highly promising contenders for next‐generation energy storage devices due to their high energy and power density, ultra‐long cycle life, and environmentally friendly nature.^[^ ^13^ ^]^ In ZIHCs, the zinc metal anode undergoes rapid surface stripping/plating, and ZIHCs have a large theoretical specific capacity of 820 mAh g^−1^.^[^ ^14^, ^15^ ^]^ Moreover, zinc metal demonstrates strong compatibility with water, and its comparatively low redox potential (−0.76 vs the standard hydrogen electrode) contributes to an expanded operating voltage window for aqueous energy storage devices. Additionally, Zn ions are more abundant and exhibit lower reactivity than monovalent ions, such as Li, Na, and K.^[^ ^16^, ^17^, ^18^ ^]^ However, Zn^2+^ in aqueous solution predominantly exists as large‐size hydrated Zn(H_2_O)_6_^2+^ (0.43 nm), which creates difficulties for the rapid insertion and expulsion of Zn^2+^ within electrode materials. Moreover, the cycle life of ZIHCs is often impeded by volume changes in the cathode induced by the insertion and expulsion of these large Zn(H_2_O)_6_^2+^ complexes. Thus, exploring advanced cathode materials with enlarged interlayer spacing and optimized void structures that can concurrently enhance zinc ion (de)insertion kinetics and structural tolerance to significant volume changes is crucial for advancing high‐performance ZIHCs.

To date, numerous transition metal oxides, including manganese‐based and vanadium‐based oxides, carbon‐based materials, MXene‐based materials, conductive polymers, and other organic materials, have been developed as promising cathodes for ZIHCs.^[^ ^19^, ^20^ ^]^ Among these, molybdenum disulfide (MoS_2_), a classical two‐dimensional (2D) transition metal sulfide, has garnered significant attention due to its natural abundance, tunable electronic properties, high specific surface area, excellent stability, and environmental friendliness.^[^ ^21^, ^22^, ^23^ ^]^ MoS_2_ with an increased interlayer spacing (≈0.62 nm) facilitates guest ions' efficient (de)insertion. However, the MoS_2_‐based cathode in ZIHCs often suffers from low storage capacity, poor rate performance, and rapid capacity degradation due to structural instability and poor electrical conductivity.^[^ ^24^, ^25^, ^26^ ^]^ To address these issues, significant efforts have been directed toward improving the performance of MoS_2_‐based cathode material by expanding MoS_2_ interlayer spacing and enhancing conductivity. For instance, Li et al.^[^ ^27^ ^]^ recently prepared MoS_2_ nanocages by intercalating nitrogen‐doped carbon motifs between the layers of MoS_2_, leading to an increase of the interlayer distance from 0.62 to 0.96 nm. As a result, the C‐MoS_2_‐N cathode exhibited an exceptional specific capacity of 247.8 mAh g^−1^ at 0.1 A/g and prolonged cyclic stability, retaining 85.6% of its capacity even after 3200 cycles in aqueous zinc ion batteries (AZIBs). Li et al.^[^ ^28^ ^]^ successfully synthesized V_2_C‐Mn composites through the intercalation of Mn^2+^, which extended the interlayer spacing and drastically increased the capacity from 350 to 500 mAh g^−1^ at a current density of 0.3 A/g for AZIBs. Geng et al.^[^ ^29^ ^]^ showed that the 1T metallic phase of MoS_2_ possesses a greater density of active sites compared to the 2H phase, leading to improved electrochemical performance. Generally, MoS_2_ exists in two phases: the metallic 1T phase and the semiconducting 2H phase. In contrast to the semiconducting 2H phase, the metallic 1T‐MoS_2_ exhibits a unique electronic structure due to its lower charge transfer resistance, distinctive metallic electronic properties, and increased interlayer distance, resulting in higher chemical activity. Unfortunately, 1T‐MoS_2_ is subject to low production, intense aging, and rapid transformation to a thermodynamically stable 2H phase. Thus, a key challenge is to simultaneously stabilize the 1T phase and further enlarge its interlayer spacing.^[^ ^30^, ^31^ ^]^ Most recently, a few people have been studying the expansion of interplanar spacing and the 1T‐2H phase transformation mechanism by intercalating organic molecules.^[^ ^32^, ^33^ ^]^ This inspires us to investigate the crucial role of ethylene glycol (EG) in expanding the interlayer spacing of MoS_2_, which is vital for enhancing ion accessibility and enabling charge storage.

In this work, we present a simple yet highly effective strategy to lower the intercalation energy barrier, significantly enhancing the intrinsic diffusivity of Zn^2+^ ions and facilitating their intercalation into layered host materials. We selected 2D MoS_2_ as a model system and engineered its structure through EG modification. This approach expanded the interlayer spacing of MoS_2_ and induced a phase transition from the semiconducting 2H phase to the highly conductive 1T phase. As a result, hydrated Zn^2+^ ion penetration was greatly improved, and charge‐transfer kinetics were substantially accelerated. The synergy of reduced intercalation energy, a uniform nanoflower‐like morphology, and the superior electrical conductivity of the 1T‐MoS_2_ phase delivered exceptional electrochemical performance, highlighting its immense potential for energy storage applications. This included an impressive capacitance of 240.5 F/g at a current density of 0.7 A/g, along with excellent cycling stability, demonstrating performance over 5000 cycles at 1.5 A/g. Furthermore, the developed ZIHC full cell achieved a high energy density of 40.42 Wh kg^−1^ at a power density of 385 W kg^−1^ while maintaining a remarkable energy density of 32.75 Wh kg^−1^ at a power density of 880 W kg^−1^.

## 2 Results and Discussion

**Figure 1:**
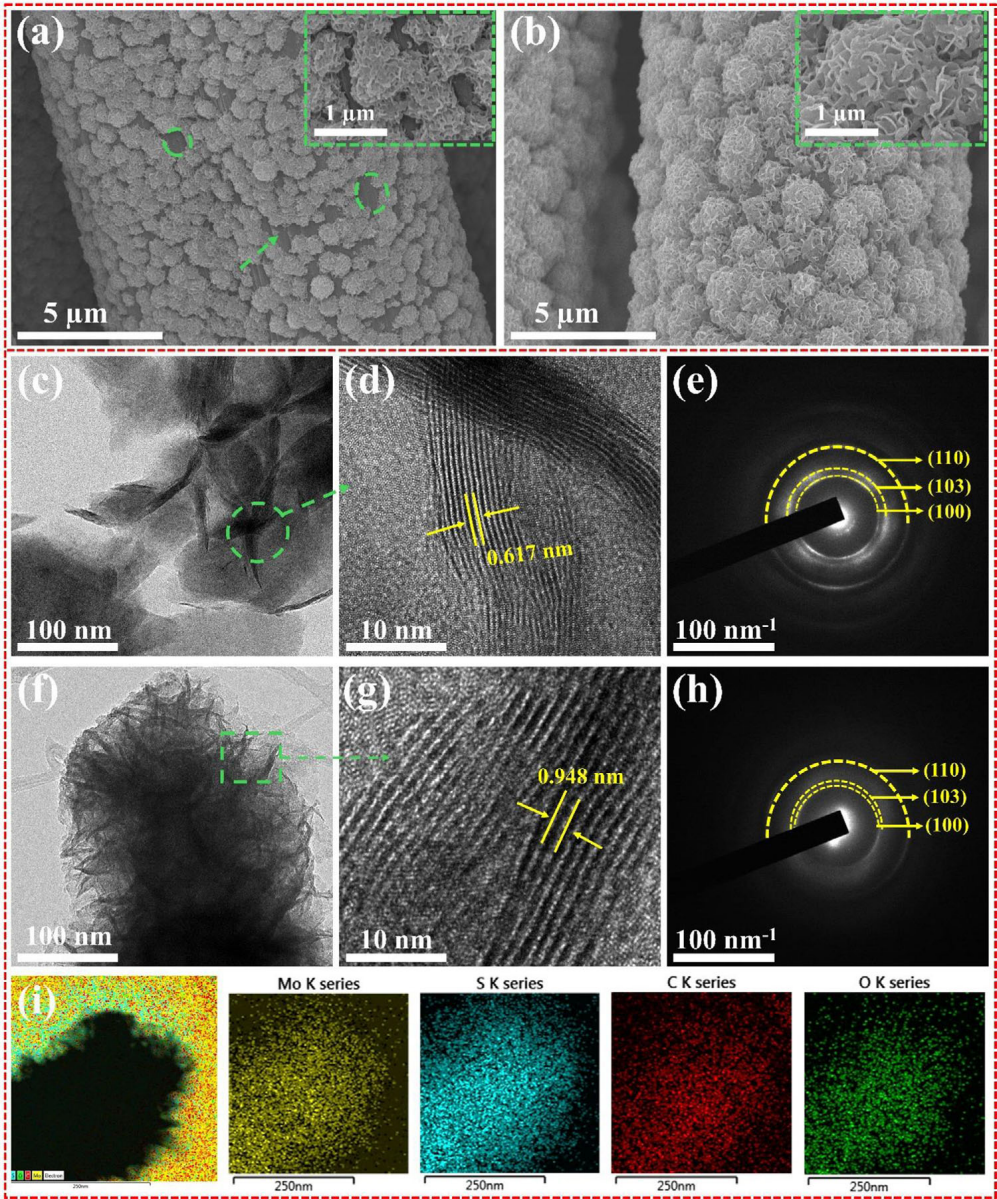
HRSEM images of a) MoS_2_ and b) EG‐MoS_2_ at low magnifications. c,d) HRTEM images, and e) SAED patterns of MoS_2_. f,g) HRTEM images, h) SAED pattern, and i) EDX mapping images of EG‐MoS_2_.

The EG‐MoS_2_ with increased interlayer spacing was synthesized via a one‐step hydrothermal process at 180 °C for 6 h. Sodium molybdate (0.01 m) and thiourea (0.02 m) were dissolved in deionized (DI) water, with 8 mL of ethylene glycol (EG) added. The prepared solution was then heated in an autoclave with carbon cloth (CC) to form EG‐MoS_2_. A similar synthesis protocol was used to prepare the pristine MoS_2_ without adding the EG. The supporting information (SI) file contains additional details about the experimental procedure. The morphological and microstructural properties of MoS_2_ and EG‐MoS_2_ were analyzed using Field‐emission scanning electron microscopy (FE‐SEM). As shown in **Figure** **1**a, pristine MoS_2_ nanoflake arrays are unevenly distributed across the surface of the CC substrate. A magnified FE‐SEM image (Figure S1a, Supporting Information) reveals that these MoS_2_ nanoflakes are aggregated and tightly packed, hence limiting the surface area available for ion absorption and desorption. Figure 1b illustrates the densely arranged, vertically aligned flake‐like array of EG‐MoS_2_ nanoflakes on CC. The high‐magnification image (Figure S1b, Supporting Information) reveals an interconnected nanoflake structure, resulting in a flower‐like morphology. This hierarchical nanostructure is favorable for preventing the stacking of S‐Mo‐S layers and facilitates the efficient transport of electrolyte ions. The addition of EG modulated the nucleation and growth mechanisms of the material, influenced by surface energy‐driven recrystallization that occurred during the hydrothermal reaction, resulting in a change in micromorphology. Notably, compared to pristine MoS_2_, EG‐MoS_2_ exhibits a more significant number of redox‐active sites readily accessible on the surface, promoting swift diffusion kinetics and a resultant high capacity. The nanostructure of the MoS_2_ nanoflower was further examined using high‐resolution transmission electron microscopy (HR‐TEM) (Figure 1c–i). Compared to pristine MoS_2_ (Figure 1c), the nanoflake size in EG‐MoS_2_ (Figure 1f) is significantly reduced, indicating that the inclusion of EG in the precursor solution affects the growth kinetics, resulting in a decrease in nanoflake size. The HRTEM images of EG‐MoS_2_ indicate that EG intercalation facilitates the miniaturization of the nanoflake, resulting in a substantial increase in edge sites. This effect is primarily attributed to the significant reduction in the surface energy of the crystal planes resulting from the presence of EG molecules.^[^ ^34^ ^]^ As shown in Figure 1h, the selective‐area electron diffraction (SAED) pattern of EG‐MoS_2_ exhibits weaker diffraction rings corresponding to the (100), (103), and (110) planes compared to those of pristine MoS_2_ (Figure 1e), indicating a lower degree of crystallinity. Moreover, the HRTEM image in Figure 1g shows that the interlayer distance of EG‐MoS_2_ was 0.948 nm, which is larger than the 0.617 nm observed in pristine MoS_2_ (Figure 1d). This expansion is attributed to the presence of interlamellar EG molecules between neighboring MoS_2_ layers, which effectively weaken the van der Waals forces. This observation further confirms the role of the EG intercalation in increasing the interlayer spacing. The enlarged interlayer significantly reduces the ion diffusion resistance and lowers the Zn^2+^ intercalation energy barrier in the electrode material, offering a viable solution for enhancing reaction kinetics and stabilizing the electrode material.^[^ ^27^, ^35^ ^]^ The energy‐dispersive X‐ray (EDX) elemental mappings of EG‐MoS_2_ are presented in Figure 1i. The EDX pattern clearly shows the uniform distributions of the Mo, S, C, and O elements on the nanoflakes, demonstrating the insertion of EG into the MoS_2_. The presence of O in the EG‐MoS_2_ may be attributed to residual oxygen in the molybdenum precursor during synthesis and sulfur vacancies that facilitate oxygen adsorption. The EDX elemental mapping for pristine MoS_2_ is shown in Figure S1c (Supporting Information), confirming the uniform distribution of Mo and S. Additionally, the nanostructure of the EG‐MoS_2_ material was evaluated using the plan and 3D views of atomic force microscopy (AFM), and the results obtained are discussed in the SI (Figure S2, Supporting Information).

**Figure 2:**
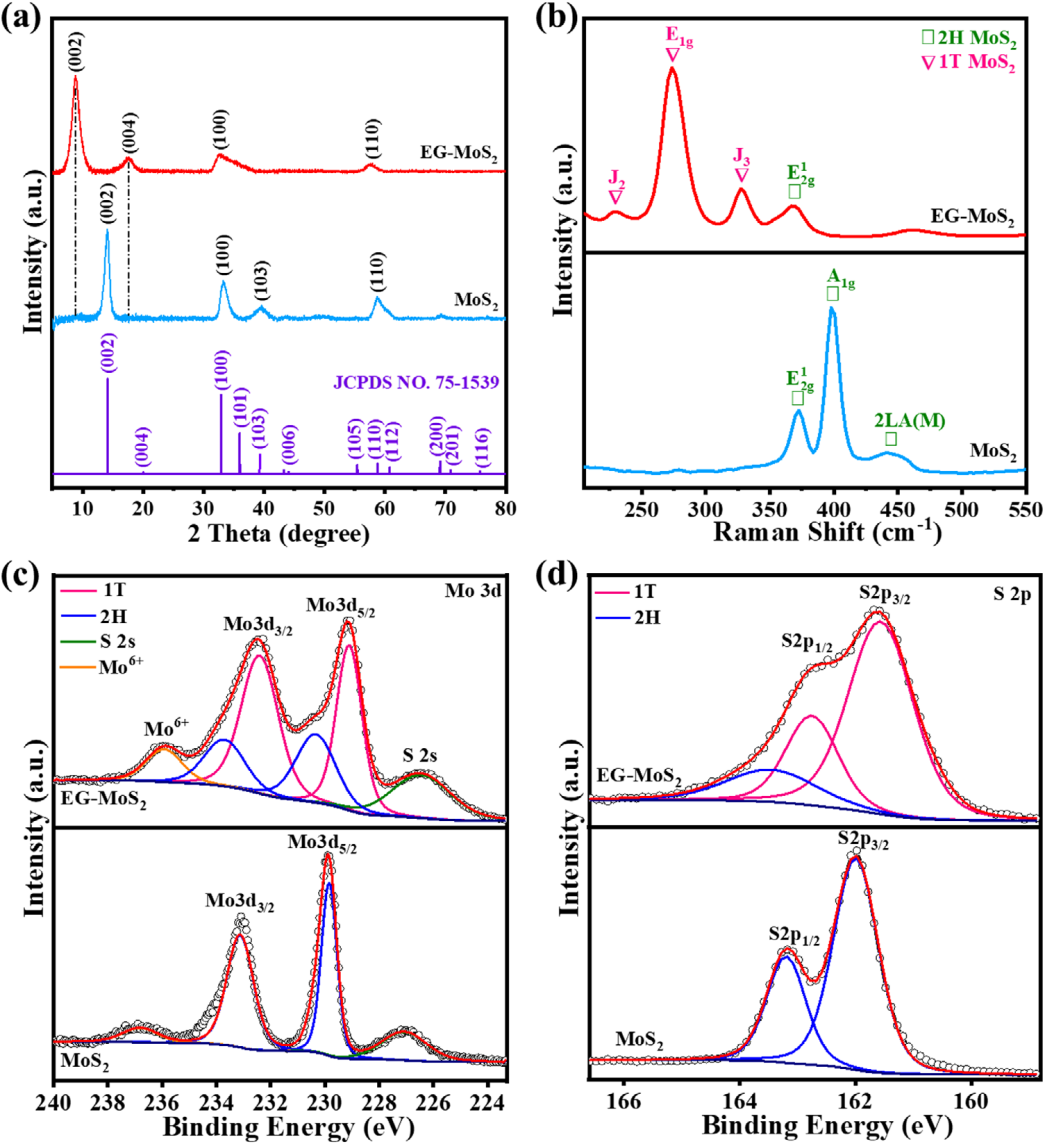
Structural characterization studies a) XRD Patterns of MoS_2_ and EG‐MoS_2_. b) Raman spectra of MoS_2_ and EG‐MoS_2_. High‐resolution spectra of MoS_2_ and EG‐MoS_2_ c) Mo *3d* and d) S *2p*.

The phase and crystal structure of MoS_2_ and EG‐MoS_2_ were further characterized using X‐ray diffraction (XRD) analysis. As shown in **Figure** **2**a, the hexagonal phase of MoS_2_ (JCPDS No. 75–1539), which belongs to space group *P*63/*mmc*, agrees well with the diffraction peaks of the pristine MoS_2_. Specifically, the reflections at 2θ = 14.1°, 33.1°, 39.4°, and 58.8° correspond to the (002), (100), (103), and (110) crystallographic planes, respectively.^[^ ^36^ ^]^ In Figure 2a, two newly generated diffraction peaks for EG‐MoS_2_, corresponding to the (002) and (004) planes, exhibit a diploid relationship and appear at 2θ of 8.9° and 17.6°, respectively.^[^ ^37^ ^]^ Meanwhile, the disappearance of the prominent (002) peak at 2θ of 14.1° indicates an expansion of the interlayer spacing relative to 2H‐MoS_2_, aligning closely with the previously reported XRD patterns characteristic of the 1T‐MoS_2_ phase.^[^ ^38^ ^]^ The interlayer spacing of 1T‐MoS_2_ nanosheets is around 0.948 nm, as computed using the Bragg equation, which exceeds that of 2H‐MoS_2_ at 0.617 nm. This value agrees well with the results obtained from HRTEM and confirms the transformation from 2H to 1T phase of MoS_2_ (Figure 1). The increased interlayer spacing in EG‐MoS_2_ is expected to lower the energy consumption and intercalation/deintercalation barrier of hydrated Zn^2+^, thereby enhancing the electrochemical performance of the material. Furthermore, Raman spectroscopy (Figure 2b) has been employed to investigate whether the insertion of organic molecules induces the phase transition of MoS_2_. The Raman spectrum of pristine MoS_2_ displays three prominent peaks at 376, 404, and 455 cm^−1^, which are attributed to the in‐plane $E_{2g}^{1}$ vibration, the vertical plane *A*_1*g*_ vibration, and the second‐order Raman scattering 2LA(M), respectively, confirming its typical 2H phase.^[^ ^39^ ^]^ In contrast, EG‐MoS_2_ demonstrates red‐shifted and less intense $E_{2g}^{1}$ and *A*_1*g*_ peak, indicating the softening of the Mo−S phonon vibrations in the basal plane due to the enlarged interlayer spacing of MoS_2_ nanosheets and the weakened interlayer forces. Furthermore, newly emerging peaks at 229.3, 284.7, and 335.0 cm^−1^, corresponding to *J*_2_, *E*_1*g*_, and *J*_3_ photon modes, respectively, provide strong evidence of a phase transformation from the 2H to 1T phase of MoS_2_ triggered by EG intercalation.^[^ ^40^ ^]^ To further investigate the presence of EG molecules within the layers of MoS_2_, Fourier‐transform infrared (FTIR) spectroscopy was conducted; results are presented in SI (Figure S3a, Supporting Information). The FTIR spectrum of the EG‐MoS_2_ confirms the incorporation of EG‐related functional groups, indicating successful intercalation of EG molecules into the MoS_2_ gallery. As displayed in Figure S3a (Supporting Information), the vibrational peaks observed at 678 and 1112 cm^−1^ in the EG‐MoS_2_ spectrum are attributed to the C─O─S and S─O bond formations, respectively.^[^ ^41^ ^]^ The C─O─S stretching vibration indicates the formation of covalent bonds between the hydroxyl groups of EG and sulfur atoms in MoS_2_, possibly via a surface functionalization process. Additionally, the S─O stretching band can indicate the partial oxidation of sulfur or the formation of intermediate sulfoxide‐type groups resulting from chemical interactions with EG molecules. These features provide strong evidence for the chemical bonding and confinement of EG molecules within the MoS_2_ galleries.^[^ ^42^ ^]^

X‐ray photoelectron spectroscopy (XPS) was conducted to analyze the elemental composition and valence state distribution of the pristine MoS_2_ and EG‐MoS_2_. The wide‐scan survey spectrum distinctly reveals the presence of Mo, S, C, and O elements within the EG‐MoS_2_ structure (Figure S3b, Supporting Information). Figure 2c,d displays the core‐level XPS profiles of Mo *3d* and S *2p* for pure MoS_2_ and EG‐MoS_2_. As shown in Figure 2c, the high‐resolution spectra of pristine MoS_2_ display two distinct peaks at 232.8 and 228.9 eV, corresponding to the Mo *3d*_3/2_ and Mo *3d*_5/2_ orbitals of Mo^4+^ in the 2H phase, respectively. Similar to MoS_2_, the high‐resolution spectra of Mo *3d* in EG‐MoS_2_ exhibit peaks at 232.4 and 229.1 eV and 233.7 and 230.3 eV, respectively, corresponding to the Mo^4+^ Mo *3d*_3/2_ and Mo^4+^ Mo *3d*_5/2_ states in the 1T and 2H phases of EG‐MoS_2_.^[^ ^43^ ^]^ This observation indicates the coexistence of 1T and 2H phases in EG‐MoS_2_. Notably, the 1T‐phase peaks exhibit a lower binding energy than the 2H‐phase peaks, likely due to the shift in the Fermi level caused by additional electron occupation of the d orbitals during the phase transition.^[^ ^44^ ^]^ The characteristic peak at 235.8 eV confirms the presence of a small amount of Mo^6+^_,_ which comes from the inevitable oxidation reaction during the XPS test of the pure MoS_2_ and EG‐MoS_2_ structures.^[^ ^45^ ^]^ In addition, one more peak was observed at 226.5 eV corresponding to the S *2s* orbital. Similarly, the high‐resolution S *2p* spectrum of EG‐MoS_2_ (Figure 2d) confirms the coexistence of the 1T and 2H phases. The 1T phase is identified by peaks at 161.8 eV (S *2p*_3/2_) and 163.1 eV (S *2p*_1/2_), while the peaks at higher binding energy (162.1 and 163.6 eV) can be attributed to 2H‐MoS_2_.^[^ ^46^ ^]^ This observation aligns with the FTIR results (Figure S3a, Supporting Information). Moreover, deconvoluted C *1s* spectra of EG‐MoS_2_ (Figure S3c, Supporting Information) exhibited four peaks at 284.6, 285.1, 286.5, and 288.3 eV, corresponding to C─C/C ═ C, C─S/C─S─Mo, C─O/C─O─Mo, and C ═ O bonds, respectively.^[^ ^33^, ^47^ ^]^ The O *1s* spectrum (Figure S3d, Supporting Information) depicts three characteristic peaks at 530.8 and 533.4 eV, attributed to C ═ O and C─OH bonds, respectively, while the primary peak at 532.1 eV corresponds to the C─O─Mo bond.^[^ ^48^, ^49^ ^]^ The detection of C─O─Mo and C─S─Mo bonds confirms the presence of chemical interactions between EG and MoS_2_,^[^ ^30^ ^]^ which are vital for maintaining the structural integrity of MoS_2_ during Zn^2+^ ionic intercalation and deintercalation processes. EG intercalation increased the interlayer spacing and distorted the trigonal prismatic coordination of the 2H phase, supplying the energy necessary to drive the phase transition of the 1T‐MoS_2_. This structural distortion introduces aberration energy, facilitating the emergence of the metastable 1T phase. Combining an expanded interlayer spacing and the highly active 1T phase enhances ion transport, increases the number of active sites for redox reactions, and ensures improved interaction with the electrolyte, ultimately boosting electrochemical performance.

**Figure 3:**
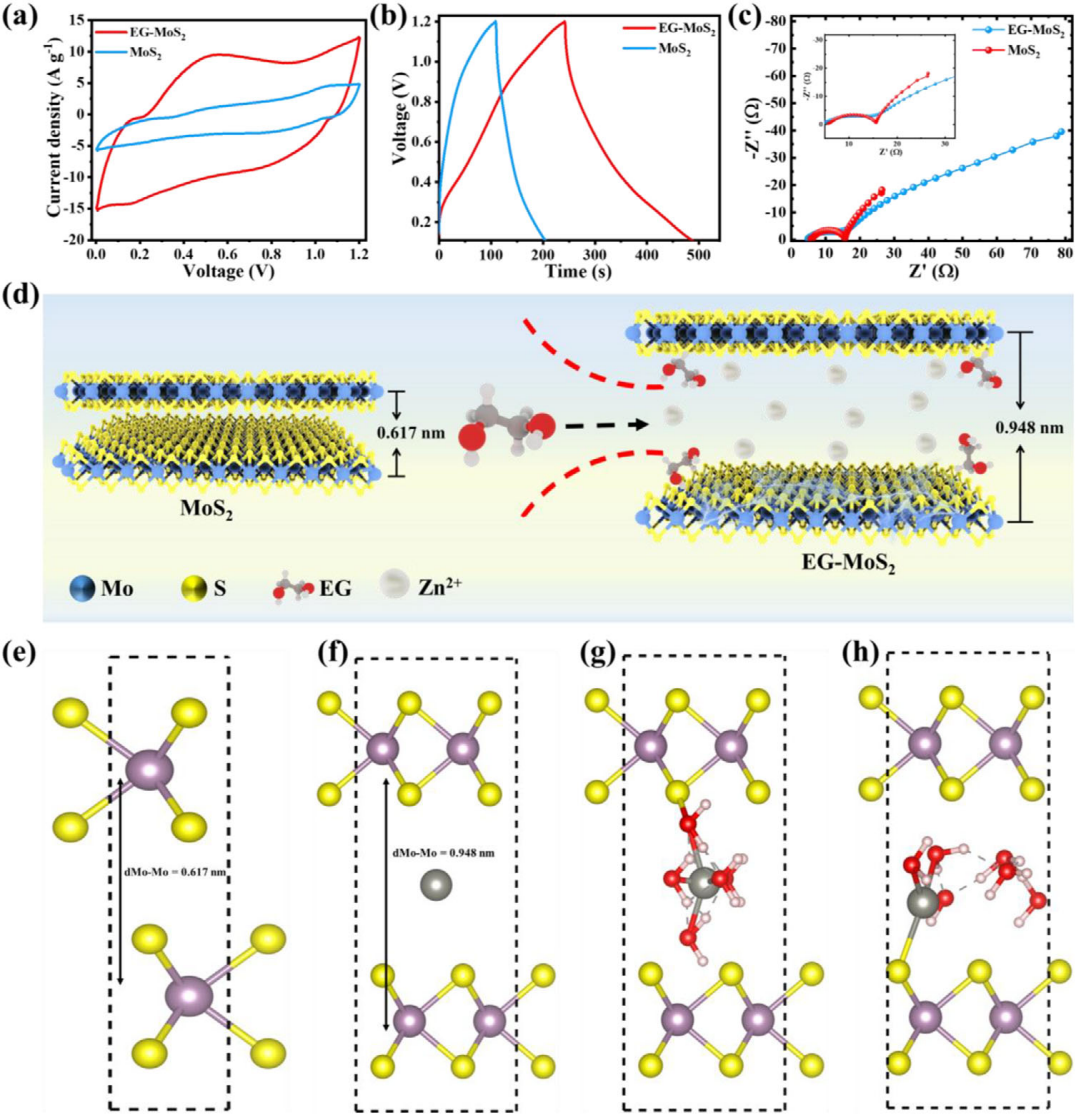
Electrochemical performance of MoS_2_ and EG‐MoS_2_ in ZIHCs within the range of 0.1‒1.2 V (vs Zn^2+^/Zn). a) Comparison of CV curves recorded at a low scan rate of 20 mV/s, b) Comparison of GCD curves at the current density of 1 A/g. c) Nyquist plots of MoS_2_ and EG‐MoS_2_. d) Schematic illustration of hydrated Zn^2+^ inserted into EG‐MoS_2_. e) Optimized relaxed geometry of the pristine MoS_2_ bilayer structure, showing the interlayer distances, f) Relaxed configuration of a Zn atom intercalated into a 2 × 2 × 1 supercell of the MoS_2_ bilayer, with the interlayer spacing increased to 0.948 nm to facilitate the intercalation, g) Initial geometry of six H_2_O molecules adsorbed onto the intercalated Zn atom in the MoS_2_ bilayer, with the interlayer spacing set to 0.948 nm, h) Relaxed geometry of the same system after structural optimization, depicting the interaction between water molecules and intercalated Zn at an interlayer spacing of 0.948 nm.

The electrochemical performance of the prepared MoS_2_ electrodes was assessed by assembling the ZIHCs, with MoS_2_ as the cathode and Zn foil as the anode in a 1 m ZnSO_4_ electrolyte. **Figure** **3**a shows the cyclic voltammetry (CV) curves of MoS_2_ and EG‐MoS_2_‐based ZIHC at a sweep rate of 20 mV s^−1^ within the potential range of 0.1‒1.2 V for comparison of the Zn^2+^ intercalation/deintercalation electrochemical reaction process. As shown in Figure 3a, all CV curves exhibit quasi‐rectangular shapes with reversible redox peaks, indicating a charge storage mechanism that involves both electric double‐layer capacitance (EDLC) and redox reactions.^[^ ^50^ ^]^ The extensive variation in CV curves underscores the crucial role of EG in enhancing Zn^2+^ storage capacity, which is further supported by the charge‐discharge curves (Figure 3b). Note that the EG‐MoS_2_ exhibits the largest integrated CV curve area compared to its control counterparts, indicating significantly improved redox kinetics and increased specific capacitance. This advancement can be ascribed to the increased fraction of the 1T phase and the enlarged interlayer spacing. These factors expose more electroactive sites and promote more efficient ion mass transfer. Figure 3b shows the galvanostatic charge‐discharge (GCD) patterns of MoS_2_ and EG‐MoS_2_ electrodes at a current density of 1 A/g. MoS_2_ and EG‐MoS_2_ exhibit triangular symmetric curves without any voltage plateau, suggesting a charge storage mechanism combining EDLC with redox reactions. Moreover, the EG‐MoS_2_ cathode exhibited the longest discharge duration, indicating its significant specific capacitance, which aligns with the CV results. The specific capacitance of pristine MoS_2_ and EG‐MoS_2_ is determined to be ≈81.8 and 223.6 F/g, respectively, at an applied current of 1 A/g. This reveals that the specific capacitance of EG‐MoS_2_ is nearly three times greater than that of pristine MoS_2_, further emphasizing its enhanced electrochemical performance. The superior performance of EG‐MoS_2_ can be mainly attributed to the presence of the highly active 1T phase and the increased interlayer spacing ensuing from the incorporation of EG, which facilitates the easy insertion and extraction of Zn^2+^ from the electrode material. Moreover, the inclusion of EG can introduce additional electrochemically active sites and structural flexibility, thereby increasing its electrochemical stability during prolonged cycles. To further investigate the enhanced charge transfer capabilities of the fabricated electrodes, electrochemical impedance spectroscopy (EIS) was performed. Figure 3c presents the comparative EIS spectra of the MoS_2_ and EG‐MoS_2_ electrodes, which were plotted and analyzed using an equivalent circuit model. Both electrodes exhibit a semicircle in the high‐frequency region, indicative of charge transfer resistance (R_ct_), and a nearly vertical line in the low‐frequency region, representing the behavior of ion diffusion. Notably, the interfacial R_ct_ of the EG‐MoS_2_ electrode was determined to be 3.02 Ω, significantly lower than that of the pristine MoS_2_ (29.17 Ω). This reduced resistance enables faster reaction kinetics and exceptional rate capability. The steep slope of the low‐frequency line further indicates the high conductivity and efficient Zn^2+^ diffusion kinetics at the electrode‐electrolyte interface. This is due to the expanded interlayer spacing of MoS_2_, which creates a favorable pathway for ion transfer and diffusion. The above electrochemical analysis confirms that pure MoS_2_ exhibits sluggish Zn^2+^ insertion kinetics, resulting in an extremely low specific capacity. However, the incorporation of EG molecules into MoS_2_ considerably enhances Zn^2+^ insertion kinetics. The schematic representation of Zn^2+^ insertion into the interlayer galleries of pristine MoS_2_ and EG‐MoS_2_ is illustrated in Figure 3d, showing the significant structural variations that affect ion transport behavior. Generally, a single Zn^2+^ ion hydrates with six water molecules in an aqueous electrolyte to form a thermodynamically stable state.^[^ ^51^ ^]^ Due to their relatively large hydrated ionic radius (≈0.43 nm), Zn^2+^ ions must undergo a desolvation process before intercalating into the host material. However, the considerable desolvation energy barrier impedes this process, and strong electrostatic interactions with the MoS_2_ framework significantly hinder the insertion of Zn^2+^ ions. In pristine MoS_2_, the distance between the two intermediate MoS_2_ layers (0.617 nm) is too narrow, making the intercalation of large Zn^2+^ hydrates unlikely or significantly hindered. Consequently, many electroactive sites remain inaccessible for charge storage.^[^ ^21^ ^]^ However, the addition of EG effectively addresses these challenges. Firstly, EG molecules expand the interplanar spacing of MoS_2_, facilitating easy diffusion of Zn^2+^ ions, even in their partially hydrated form. This expansion mainly reduces diffusion resistance and improves the energetic barrier associated with ion desolvation. Furthermore, as documented in previous studies, EG can act as a stabilizing “pillar” during the Zn^2+^ intercalation and deintercalation processes, thereby maintaining the structural integrity of the MoS_2_ framework.

We performed density functional theory (DFT) calculations to explore the intercalation of hydrated zinc ions between MoS_2_ layers. The computational details of DFT calculations are provided in the SI file. Our study began by fully relaxing a MoS_2_ bilayer, yielding optimized lattice parameters of a = b = 0.316 nm and an interlayer Mo‒Mo distance (d_Mo‐Mo_) of 0.617 nm, which aligns well with previous experimental findings (d_Mo‐Mo_ = 0.62 nm)^[^ ^52^, ^53^, ^54^ ^]^ and other DFT results.^[^ ^55^, ^56^ ^]^ The optimized MoS_2_ bilayer geometry is depicted in Figure 3e. Metal ion intercalation into the van der Waals gaps of MoS_2_ can profoundly alter its structural and electronic properties, influencing interlayer spacing and interaction strength‐critical factors for energy storage applications. For pristine MoS_2_, the interlayer distance between sulfur atom layers (d_S‐S_) is found to be 0.305 nm, which is too narrow to accommodate large hydrated zinc ions (Zn(H₂O)_6_^2+^) with a diameter of ≈0.55 nm. It has been observed that small increases in interlayer spacing (0.01–0.08 nm) are sufficient to intercalate smaller alkali metals, such as Li^+^, Na^+^, and K^+^.^[^ ^50^, ^51^ ^]^ However, significantly larger spacing adjustments may be required to accommodate hydrated zinc ions.^[^ ^21^ ^]^ We introduced a Zn atom into the MoS_2_ bilayer unit cell at varying interlayer spacings (in increments of 0.05 nm) and performed constrained relaxation, keeping the interlayer distance fixed. The intercalation energy of Zn was calculated to be +0.78, −0.54, −0.72, −0.93, −0.98, and −0.94 eV for interlayer distances of 0.643, 0.690, 0.740, 0.790, 0.840, and 0.948 nm, respectively. These results indicate that the intercalation of Zn is energetically favorable for all interlayer spacings except for the relaxed MoS_2_ bilayer spacing (d = 0.643 nm). A small increase of ≈0.05 nm in interlayer spacing is thus required to facilitate Zn intercalation. Next, we investigated the adsorption of water molecules on the intercalated Zn. Interestingly, even for the largest interlayer spacing (d = 0.948 nm), the adsorption of a single H_2_O molecule was found to be energetically unfavorable (adsorption energy of +0.4 eV). We then examined a larger system, intercalating one Zn atom into a 2 × 2 × 1 supercell of the MoS_2_ bilayer. In this case, the intercalation energy of the Zn atom is found to be −0.75 eV at an interlayer distance of 0.948 nm, with the optimized geometry shown in Figure 3f. Notably, when Zn concentration was reduced (one Zn atom per 2 × 2 × 1 supercell), water adsorption became energetically favorable, with the adsorption energy of the first H_2_O molecule being −0.33 eV at d = 0.948 nm.

To further assess whether the MoS_2_ bilayer can accommodate large zinc hydrate ions, we examined the adsorption of six H_2_O molecules on the Zn‐intercalated 2 × 2 × 1 MoS_2_ bilayer system. The average adsorption energy of six H_2_O molecules adsorbed on the Zn‐intercalated 2 × 2 × 1 MoS_2_ bilayer system was found to be −0.26 eV/H_2_O. We found that five of the six water molecules adsorb in molecular form when the interlayer spacing is increased to 0.948 nm. Of these, three H_2_O molecules form Zn–O bonds with lengths of 0.198, 0.199, and 0.205 nm, while two H_2_O molecules exhibit longer Zn─O distances of 0.304 and 0.427 nm. The sixth H_2_O molecule dissociates into OH^−^ and H^+^ ions. The initial and relaxed geometries of the system with six H_2_O molecules are shown in Figure 3g,h, respectively. Our findings suggest that a zinc hydrate complex can be accommodated with five water molecules and one dissociated water molecule when the interlayer spacing of the MoS_2_ bilayer is increased to 0.948 nm.

**Figure 4:**
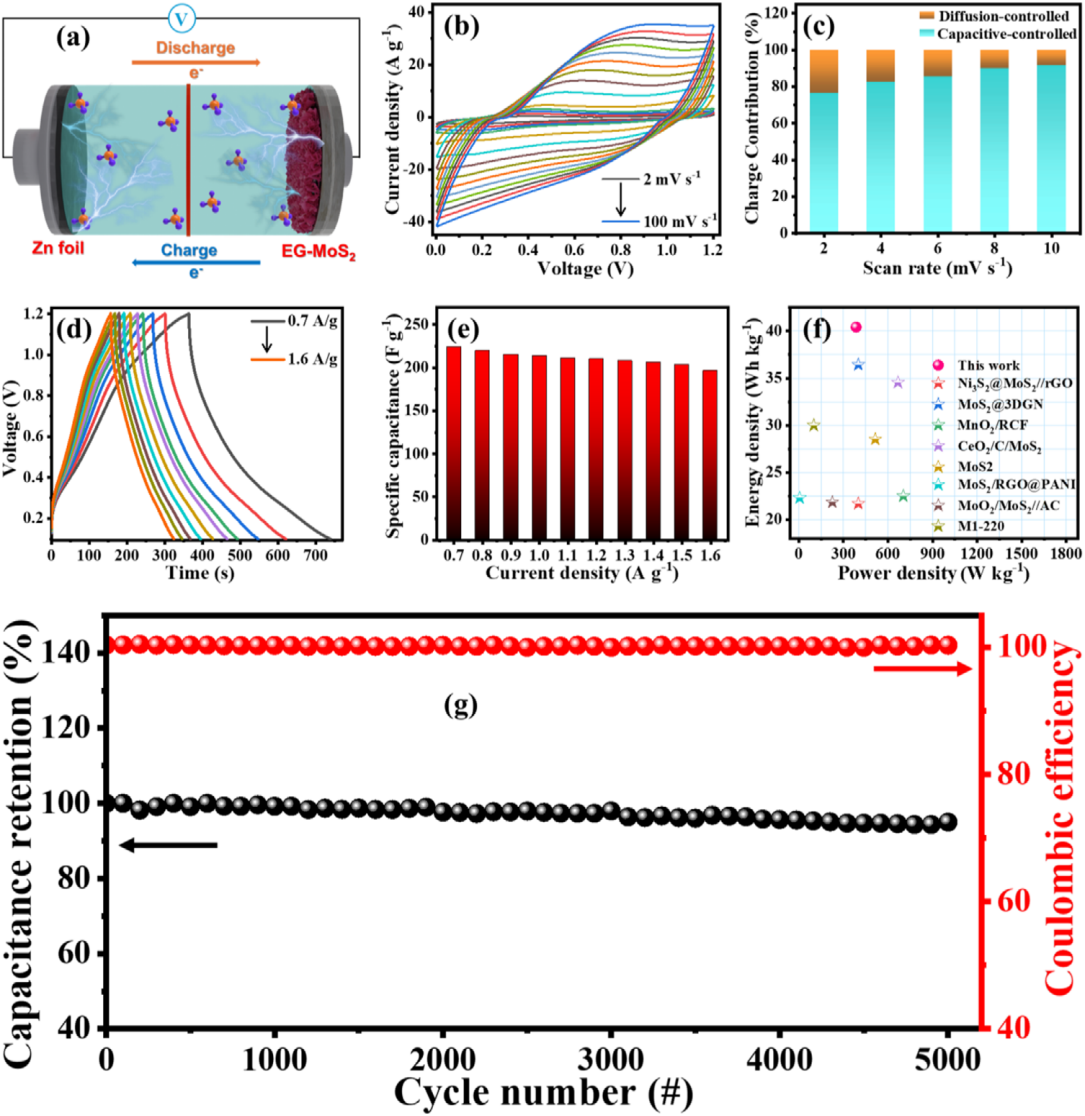
a) Schematic presentation of ZIHCs, b) CV curves of EG‐MoS_2_, c) capacitive and diffusive charges stored at various scan rates for EG‐MoS_2_, d) GCD curves of EG‐MoS_2_, e) specific capacitance of EG‐MoS_2_, f) Ragone plot and e) long‐term cycling stability of EG‐MoS_2_.

**Figure** **4**a demonstrates the schematic diagram of an assembled ZIHC, which consists of EG‐MoS_2_ as the cathode, a Zn foil as the anode, and a 1 m ZnSO_4_ as the electrolyte. The Zn^2+^ storage kinetics of the EG‐MoS_2_ electrode were investigated using CV at scan rates varying from 2 to 100 mV s^−1^. As shown in Figure 4b, the CV curves maintain their shape and redox peak positions across the full range of sweeping rates, indicating the outstanding rate performance of EG‐MoS_2_ mentioned earlier. With increasing scan rates, the anodic and cathodic peaks shift toward higher and lower potentials due to increased ion diffusion hysteresis. During continuous scanning, the peak area progressively expanded with rising current density, which can be attributed to the gradual stabilization of the electrode structure during the initial charge‐discharge cycles. For a battery system or a capacitor system, the charge storage mechanism is closely related to the rate of the charge/discharge process.^[^ ^57^ ^]^ Therefore, the relationship between peak current (*i*) and scan rate (*ν*) at lower sweep rates can be depicted by the equation below:^[^ ^58^, ^59^, ^60^ ^]^

(1) $$i=av^{b}$$

(2) $$\log\left(i\right)=b\log\left(v\right)+\log\left(a\right)\;$$

where *i* represents the current density of the cathodic or anodic peak observed in the CV curves, while *ν* denotes the scan rate (ranging from 2 to 10 mV s^−1^). The parameters *a* and *b* are two adjustable constants, corresponding to the intercept and slope of the log(*i*) versus log(*ν*) plot. The value of *b* determines the charge storage mechanism, typically ranging between 0.5 and 1. Specifically, when exponent *b* approaches 0.5, it indicates that the redox reaction is diffusion‐controlled, whereas *b* = 1 signifies a surface‐controlled capacitive charge storage process. For the EG‐MoS_2_ composite electrode, the *b* values derived from the cathodic and anodic peaks are 0.88 and 0.84, respectively, as depicted in Figure S4 (Supporting Information). These values indicate hybrid storage behavior involving capacitive and diffusion‐controlled processes, with capacitive control playing a predominant role. The exact contributions from the capacitive and diffusion control methods in the CV plots at distinct scan rates were also calculated using the following equations.

(3) $$i\;\left(V\right)=k_{1}v+k_{2}v^{1/2}\;$$

(4) $$\frac{i\left(V\right)}{v^{1/2}}=k_{1}\;v^{1/2}+k_{2}$$

In the equation provided, *k*_1_and *k*_2_ are constants, where *k*_1_ *v* representing the capacitive and *k*_2_ *v* ^1/2^ corresponding to the diffusion‐controlled contributions of the electrode reactions. Figure 4c illustrates ratios of capacitive and diffusion‐controlled contributions at various scan rates. As the sweep rates increased from 2 to 10 mV s^−1^, the diffusion‐controlled contribution of the EG‐MoS_2_ cathode decreased from 23.5% to 8.4%, indicating that the capacitive contribution is enhanced with increasing scan rates. At a scan rate of 10 mV s^−1^, the capacitive contribution reaches 91.5%, suggesting that a capacitive process predominantly dominates the energy storage behavior of the ZIHC and demonstrates the rapid electrochemical kinetics of EG‐MoS_2_. The predominant capacitive behavior is primarily attributed to the expanded interlayer spacing and improved electrical conductivity of 1T‐MoS_2_, facilitated by EG intercalation.

The GCD graphs of the EG‐MoS_2_ electrode at different current densities from 0.7 to 1.6 A/g are displayed in Figure 4d. The GCD profiles exhibit nearly symmetric triangular shapes with trivial IR drops during discharge, suggesting efficient and rapid charge transfers in the EG‐MoS_2_. The charge and discharge plots of EG‐MoS_2_ indicate lower diffusion resistance and enhanced charge‐storage kinetics in the system. Figure 4e illustrates the specific capacitance values derived from the discharge curves recorded at various current densities. Notably, the electrode achieves a maximum specific capacitance of 240.5 F/g at a current density of 0.7 A/g, highlighting the enhanced charge adsorption and Zn^2+^ storage capacity achieved by the EG‐MoS_2_ composite. Even as the current density rises from 0.7 to 1.6 A/g, the EG‐MoS_2_ electrode exhibited an excellent capacitive retention of 90.2%. The obtained results exceed the superiority of metal sulfide electrodes reported in recent years.^[^ ^61^ ^]^ To further evaluate the electrochemical features of the cell, the energy and power densities of EG‐MoS_2_ ZIHC were also determined from the charge‐discharge data (Figure 4f). The Ragone plot indicates that the EG‐MoS_2_ ZIHC delivers a superior energy density of 40.42 Wh kg^−1^ at a power density of 385 W kg^−1^. Even when the power density is increased over two‐fold to 880 W kg^−1^, the device still retains a commendable energy density of 32.75 Wh kg^−1^. These results surpassed those of many previously reported metal sulfide‐based electrodes and even outperformed those of metal oxides studied for ZIHCs and SCs.^[^ ^62^, ^63^, ^64^, ^65^, ^66^, ^67^, ^68^, ^69^, ^70^ ^]^ The comparative results are shown in Table S1 (Supporting Information). Furthermore, the long‐term cycling stability of ZIHCs was assessed, as it is a crucial feature of energy‐storage devices and essential for commercial viability. The cycling performances of the as‐fabricated ZIHC were tested at a current density of 1.5 A/g (Figure 4g). The ZIHC demonstrated excellent cycling stability and durability, retaining 94.9% of its capacity after 5000 charge‐discharge cycles. Additionally, the coulombic efficiency consistently approached 100% during the entire cycling process, highlighting excellent electrochemical reversibility. Such performance confirms the superior kinetic properties and stable electrochemical behavior of the EG‐MoS_2_ cathode, making it a highly promising candidate for next‐generation zinc‐ion energy storage systems. The electron and ion transport characteristics of EG‐MoS_2_ electrodes have been meticulously investigated through EIS. Figure S5 (Supporting Information) displays the Nyquist plot and the equivalent circuit diagram, providing a comprehensive visualization of impedance behavior. The equivalent circuit diagram comprises main components, including equivalent series resistance (R_s_), charge‐transfer resistance (R_ct_), constant phase element (C_PE_), and Warburg impedance (Z_w_). In the Nyquist plot, the semicircle in the high‐frequency region corresponds to the R_ct_ at the cathode‐electrolyte interface. As presented in Figure S5 (Supporting Information), the R_ct_ of the EG‐MoS_2_ ZIHC device is 8.09 Ω, demonstrating the better transfer kinetics property due to its expanded interlayer spacing and excellent structural stability. The exceptional charge transfer and ion‐diffusion kinetics of the ZIHC are primarily due to the excellent electrical conductivity of the electroactive material, which enables efficient electron pathways, facilitates easy electrolyte penetration, and provides readily accessible active sites for zinc‐ion storage. Ultimately, these findings contribute to advancing the development of high‐performance and durable zinc‐ion storage systems for large‐scale energy applications.

**Figure 5:**
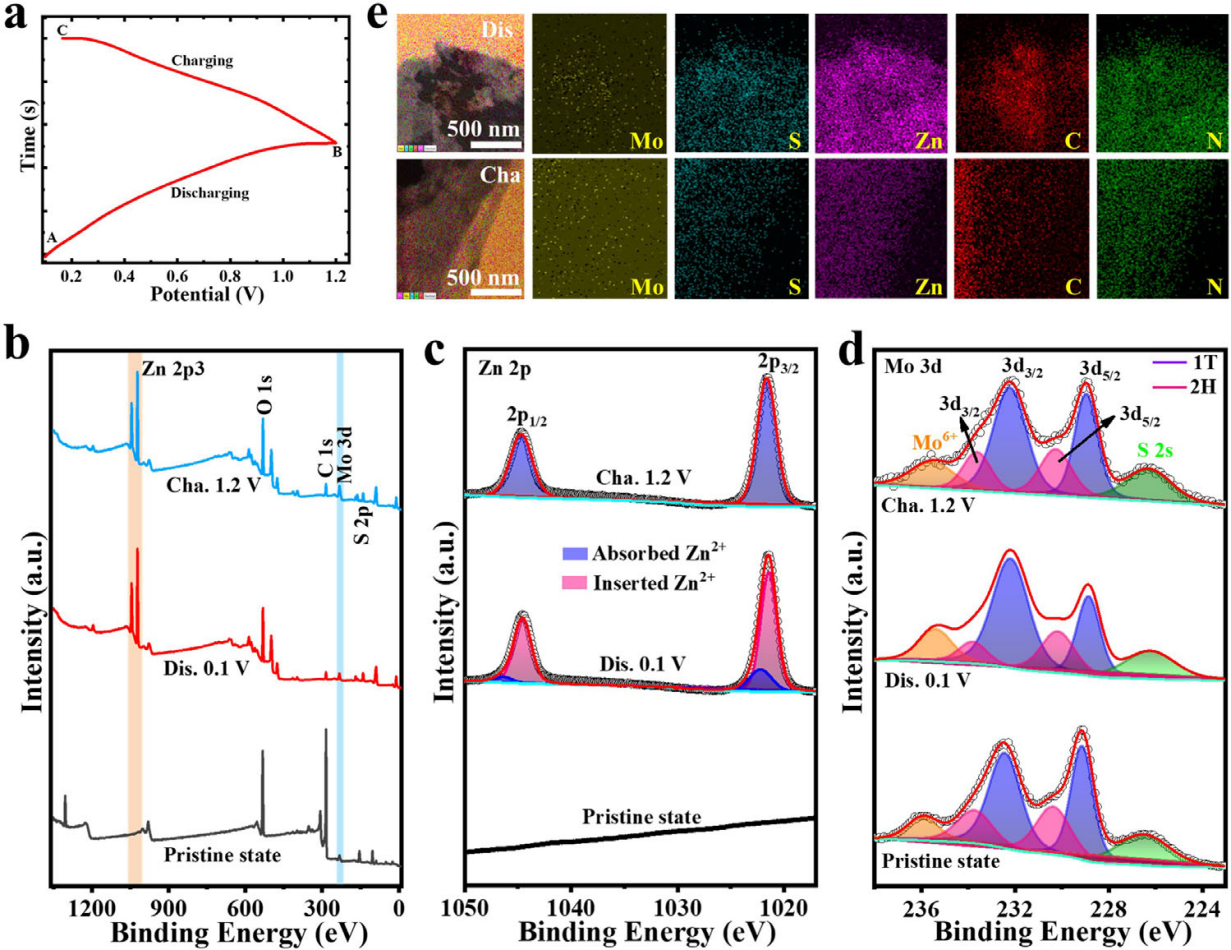
a) Selected voltage points in the typical discharge/charge curves. Ex situ XPS and EDX mapping characterizations of the EG‐MoS_2_ cathodes at different discharge/charge states b) Ex situ XPS survey spectra of EG‐MoS_2_. Ex situ study of high‐resolution c) Zn *2p* and d) Mo *3d* spectra of the pristine, fully discharged, and charged EG‐MoS_2_ electrode. e) The elemental mapping images of the fully discharged and charged EG‐MoS_2_ cathode.

To elucidate the Zn‐storage mechanism, the chemical state and elemental configuration of pristine, discharged, and charged EG‐MoS_2_ were analyzed by ex situ XPS and HRTEM‐EDX mapping characterizations (**Figure** **5**). The surface chemical transformation of the EG‐MoS_2_ cathode at distinctive charge‐discharge states (as indicated by the points presented in the typical first discharge and second charge curves) was examined through ex situ XPS (Figure 5a). The full survey spectrum of the cathode at various charge‐discharge stages is shown in Figure 5b, revealing evident changes in the Zn *2p* and Mo *3d* spectra. Figure 5c displays the high‐resolution Zn *2p* spectra of the EG‐MoS_2_ in different states. In the pristine state, no Zn signals are observed in the Zn *2p* XPS spectrum. However, upon discharging to 0.1 V, the deconvoluted spectrum reveals two pairs of Zn *2p* signals, which correspond to Zn^2+^ absorbed on the cathode surface and Zn^2+^ inserted into the EG‐MoS_2_ structure. The successful Zn^2+^ insertion is further confirmed by the identical distribution of Mo, S, Zn, C, and O elements in the fully discharged electrode, as shown in Figure 5e. After charging to 1.2 V, only a single pair of the Zn *2p* signals remains, which is attributed to the surface adsorption of the electrolyte. The Zn element mapping of the charged electrode (Figure 5c) also shows a significant reduction in Zn content, confirming that the reversible Zn^2+^ insertion and extraction process primarily drives the energy storage capability of the EG‐MoS_2_ cathode. Figure 5d displays the Mo *3d* spectrum of EG‐MoS_2_. An XPS peak at 226.5 eV is assigned to the S *2s* orbital. In the Mo *3d* core‐level spectrum of pristine‐state, three distinct peaks are observed, after deconvolution, corresponding to valence states of Mo^4+^ (*3d*_3/2_: 232.3 eV, *3d*_5/2_: 229.3 eV), Mo^5+^ (*3d*_3/2_: 233.2 eV, *3d*_5/2_: 230.3 eV), and Mo^6+^ (*3d*_3/2_: 235.4 eV).^[^ ^71^ ^]^ Upon fully discharged state, the prominent characteristic peaks of Mo^4+^ and Mo^5+^ were noticeably suppressed. These findings showed that Zn^2+^ ions were inserted and extracted from the EG‐MoS_2_ cathode during charging and discharging. The above results conclude that the EG‐MoS_2_ cathode is a promising candidate for zinc ion storage. Firstly, the intercalation of EG promotes the phase transition of MoS_2_ from the 2H to the 1T phase, resulting in an abundance of edge‐active sites and surfaces. This transition enhances Zn^2+^ storage by providing more efficient channels for ion and electron transport. Secondly, the expanded interlayer spacing and structural disorder could facilitate the kinetics of Zn^2+^ intercalation during electrochemical reactions, resulting in a higher capacitance contribution and greater zinc storage capacity. Lastly, the 2D nanoflower‐like morphology of EG‐MoS_2_ increases the electrode's specific surface area, facilitating three‐dimensional ion diffusion and transport pathways that facilitate fast electrochemical reactions.

## 3 Conclusion

In summary, we developed a single‐step, tunable approach to synthesize MoS_2_ with expanded interlayer galleries, thereby facilitating the rapid and efficient intercalation and deintercalation of hydrated Zn^2+^ ions during charge‐discharge cycles. Experimental results reveal that the superior electrochemical performance of the EG‐MoS_2_ electrode stems from the intercalation of EG molecules within the MoS_2_ layers. This process expands the interlayer spacing, regulates the transition to the 1T phase, and exposes additional active sites, facilitating the efficient intercalation and deintercalation of Zn^2+^ ions. Simultaneously, the enhanced electronic conductivity accelerates charge transport kinetics, further boosting overall performance. Thanks to the embedded EG, the layer spacing of the EG‐MoS_2_ is extended from 0.617 to 0.948 nm. DFT calculations demonstrated that the expanded interlayer spacing significantly reduces the intercalation energy barrier for hydrated Zn^2+^, thereby boosting the material's energy storage capacity. As a result, the obtained EG‐MoS_2_ nanosheet electrode demonstrates a specific capacity of 240.5 F/g at 0.7 A/g as a cathode for ZIHC. Furthermore, the ZIHC showed remarkable energy and power characteristics, achieving a high energy density of 40.42 Wh kg^−1^ at a power density of 385 W kg^−1^. Notably, even at an elevated power density of 880 W kg^−1^, they maintained a commendable energy density of 32.75 Wh kg^−1^, highlighting their excellent energy retention and rapid charge‐discharge capabilities. Interestingly, it showed excellent cycling performance, with a capacitive retention of 94.9% after 5000 cycles and a coulombic efficiency of 100%. This work presents an effective strategy for engineering MoS_2_‐based cathode materials with superior electrochemical performance, paving the way for next‐generation high‐performance ZIHCs.

## Conflict of Interest

The authors declare no conflict of interest.

## Supporting information



Supporting Information

## Data Availability

The data that support the findings of this study are available from the corresponding author upon reasonable request.
