# Supplementary material for: One‐Step Tunable MoS2 with Enhanced Zn2+ Diffusion for High‐Energy Zinc‐Ion Hybrid Capacitors
Source: Adv Sci (Weinh). 2025 Sep 23;12(41):e06467. doi: 10.1002/advs.202506467 (PMC12591203; doi:10.1002/advs.202506467)
Supplement: Supplementary file 1 — Supporting Information [file ADVS-12-e06467-s001.docx]

**One-Step Tunable MoS_2_ with Enhanced Zn^2+^ Diffusion for High-Energy Zinc-Ion Hybrid Capacitors**

*Harshitha B. Tyagaraj,* *Vikram Mahamiya, Supriya J. Marje, Gagankumar Sakleshpur Kumar, Shalmali R. Burse, Swapnil R Patil, Ebrahim Al Hajri, Nilesh R. Chodankar*, Yun Suk Huh*, Young Kyu Han**

**Ms. Harshitha B. Tyagaraj, Dr. Supriya J. Marje, Mr. Gagankumar Sakleshpur Kumar, Ms. Shalmali R. Burse, Prof. Young-Kyu Han** ([ykenergy@dongguk.edu](mailto:ykenergy@dongguk.edu))

Department of Energy and Materials Engineering, Dongguk University-Seoul, Seoul 04620, Republic of Korea

**Dr. Vikram Mahamiya**

Condensed Matter and Statistical Physics Section, The Abdus Salam International Centre for Theoretical Physics (ICTP), Strada Costiera 11, 34151 Trieste, Italy

**Prof. Ebrahim Al Hajri, Dr. Nilesh R. Chodankar** ([nilesh.chodankar@ku.ac.ae](mailto:nilesh.chodankar@ku.ac.ae))

Mechanical and Nuclear Engineering Department, Khalifa University of Science and Technology, 127788, Abu Dhabi, United Arab Emirates

**Dr. Swapnil R. Patil, Prof. Yun Suk Huh** ([yunsuk.huh@inha.ac.kr](mailto:yunsuk.huh@inha.ac.kr))

Department of Biological Science and Bioengineering, Nanobio High-Tech Materials Research Centre, Inha University, Incheon, 22212, South Korea

**Experimental details**

**Synthesis of EG-MoS_2_**

Analytical-grade chemicals and reagents were used without further purification. Before the experiment, the carbon cloth (CC) substrate was treated with a concentrated HNO_3_ and HCl solution for 1 hour and then cleaned with deionized (DI) water several times. In a typical synthesis, 0.01 M sodium molybdate (Na_2_MoO_4_), 0.02 M thiourea (H_2_NCSNH_2_), and 8 mL of ethylene glycol were dissolved in 70 mL of deionized (DI) water and stirred for 30 minutes to form a homogeneous solution at room temperature. The solution obtained and a piece of cleaned CC substrate (1.5 cm × 3 cm) were transferred to a Teflon-lined autoclave. A hydrothermal treatment at 180 °C for 6 hours was carried out to generate the final product. The CC with the as-grown EG-MoS_2_ was rinsed with deionized (DI) water and dried at 60 °C for 12 hours in a vacuum oven. The mass loading of as-synthesized EG-MoS_2_ on CC was 2 mg cm^-2^. For comparison, Pure MoS_2_ with a mass loading of 2 mg cm^-2^ was fabricated using the same synthetic procedure without adding EG.

**Structural and surface characterization**

The morphological characteristics and nanostructures of the prepared samples were investigated using a field-emission scanning electron microscope (FE-SEM, HITACHI S-4800, Japan) and a high-resolution transmission electron microscope (HR-TEM, JEM-2100F, JEOL, Japan), respectively. The phase and crystal structures of the prepared samples were examined by X-ray diffraction (XRD, X'Pert-PRO MRD, Philips, The Netherlands) using Cu Kα irradiation. Raman spectra were recorded using a laser Raman spectrometer (Raman, FEX, NOST, Republic of Korea) at an excitation wavelength of 532 nm. X-ray photoelectron spectroscopy (XPS) was conducted using a Thermo Fisher Scientific K-alpha to analyze the surface elements and their chemical bond states. Fourier transform infrared (FTIR) spectra were obtained using a JASCO FTIR spectrometer and the KBr pellet technique, spanning the range from 800 to 200 cm^−1^, to identify the surface functional groups.

**Electrochemical measurements**

The electrochemical performance of the as-prepared materials was measured on a Zive SP6 electrochemical workstation in a typical two-electrode system. Cyclic voltammetry (CV), galvanostatic charge-discharge (GCD), and Electrochemical impedance spectroscopy (EIS) were performed to characterize the electrochemical behavior of the prepared electrodes. The CV and GCD measurements were conducted in the potential range of 0.1−1.2 V (vs Zn^2+^/Zn). EIS experiments were performed within a frequency range of 10 to 100 mHz at an open circuit potential with an amplitude of 10 mV. The cycling stability of the ZIHCs is measured using a battery test system (Cyclier, WBCS3000M1). In this case, the MoS_2_-based ZIHCs are assembled using the above MoS_2_ materials as the cathode and a high-purity Zn plate as the anode, with Whatman GF/A glass microfiber as the separator in a 1 M ZnSO_4_ electrolyte.

**Formulae used for calculations**

The specific capacitance (Cs) (F g^-1^) was calculated from the discharge curve using the following formulae,

$$C_{s}=\frac{I \times\Delta t}{w \times\Delta V} (1)$$

Where I, Δt, w, and ΔV are current density, discharge time, mass of active material, and voltage window, respectively.

Specific energy (E, Wh kg^-1^) and specific power (P, W kg^-1^) were calculated using the following equations.

$$E\text{ = }\frac{0.5 \times C_{s} \times{\Delta V}^{2}}{3.6} (2)$$

$$P=\frac{E \times3.6}{\Delta t} (3)$$

**Computational Details**

We carried out first-principles density functional theory (DFT) calculations for structural optimizations using the Perdew‒Burke‒Ernzerhof (PBE) ^[1]^ exchange‒correlation functional within the generalized gradient approximation (GGA) framework, as implemented in the Vienna ab initio simulation package (VASP).^[2–5]^ A plane-wave energy cutoff of 520 eV was applied for the expansion of the wavefunctions. The hexagonal structure of MoS_2_ (space group P6_3_/mmc) was selected from the ICSD structural database,^[6]^ and bilayers of two MoS_2_ units and 2×2×1 supercell of these units were used to model the intercalation of hydrated zinc ions between the MoS_2_ layers. The Brillouin zone was sampled with a Γ-centered 8×8×2 k-point grid. Strict convergence thresholds were set to 10^-8^ eV and 10^-7^ eV/Å for total energy and atomic forces convergence, respectively, ensuring high precision in both the geometries and total energies. Van der Waals interactions were accounted for using Grimme’s DFT-D3 dispersion correction method^[7]^ to capture long-range dispersion forces accurately.

Figure S1 HRSEM images of (a) MoS_2_ and (b) EG-MoS_2_ at high magnifications. (c) EDX mapping images of MoS_2_.

Atomic force microscopy (AFM) is used to obtain a high-resolution 2D topography and 3D mapping of the material’s surface, providing nanodimensional data on the structure. To verify the nanostructure of EG-MoS_2_, the plan and 3D views of AFM images are displayed in Figures S2a & b. Since EG-MoS_2_ nanoflakes have a diameter of ~ 3 µm and a thickness of about 30 nm, it is confirmed that EG-MoS_2_ is a 2D material.

Figure S2 AFM image displays 2D and 3D surface profiles of EG-MoS_2_.

Figure S3 (a) FTIR spectra of MoS_2_ and EG-MoS_2_. (b) Full scan XPS profiles of MoS_2_ and EG-MoS_2_. High-resolution (c) C *1s* and (d) O *1s* XPS profiles of EG-MoS_2_.

Figure S4 The plot of log (current density) *versus* log (scan rate) to determine the ‘b’ value of the EG-MoS_2_ electrode.

Figure S5 Nyquist plots (inset: fitted circuit) of EG-MoS_2_ electrode.

Table S1 The performance evaluation of EG-MoS_2_ ZIHCs device with past reported metal-ion capacitors.

| **Electrode materials** | **Electrolyte** | **Capacitance** (F g^-1^) | **Energy density** (Wh kg^-1^) | **Power density** (W kg^-1^) | **Stability** | **Ref.** |
| --- | --- | --- | --- | --- | --- | --- |
| EG-MoS_2_ | 1 M ZnSO_4_ | 240.5 | 40.42 | 385 | 94.9 % (5000) | Present study |
| MoS_2_-160 | 3 M Zn(CF_3_SO_3_)_2_ | 168 |  |  |  | ^[8]^ |
| MoS_2_/Graphene | Zn(CF_3_SO_3_)_2_ | 141.6 | 32.5 |  | 88.2 % (1800) | ^[9]^ |
| MoS_2_/Mo_2_CT*_x_* | 1 M NaClO_4_ | 207.4 | 23.3 | 3500 | 76.8 % (5000) | ^[10]^ |
| MoS_2_@VZnS | 1 M KOH | 285.4 | 39.54 | 2462.35 | 84.53 % (10000) | ^[11]^ |
| 2D MoS_2_@rGO | 1 M Na_2_SO_4_ | 81 | 36.5 | 9 | 88 % (5000) | ^[12]^ |
| MoS_2_/PEI-GO | 2 mol L^-1^ Na_2_SO_4_ | 42.9 | 19.3 | 4500 | 91.3 % (8000) | ^[13]^ |
| CuS/PANI@MoS_2_ | 2 mol L^-1^ H_2_SO_4_ | 166.7 | 39.1 | 659.9 | 85.5 % (5000) | ^[14]^ |
| NiS_2_@MoS_2_ | 6 M KOH | 103.1 | 37.2 | 0.8 | 80.3 % (10000) | ^[15]^ |
| s-MoS_2_//CNS | 1 M Na_2_SO_4_ | 231 | 7.4 | 3700 |  | ^[16]^ |
| MoS_2_@TiO_2_ | 1 M Na_2_SO_4_ | 210 | 21 | 1350 | 98 % (2000) | ^[17]^ |
| MoS_2_ | 0.5 M TEABF_4_ | 14.75 | 18.43 | 1125 | 91.2 % (5000) | ^[18]^ |
| Co-exfoliated GNP/MoS_2_ | 1 M Na_2_SO_4_ | 80 | 36.23 | 1799.75 | 92.87 % (2100) | ^[19]^ |

**Reference**

[1] J. P. Perdew, K. Burke, M. Ernzerhof, *Phys. Rev. Lett.* **1996**, *77*, 3865.

[2] R. A. Vargas-Hernández, *J. Phys. Chem. A* **2020**, *124*, 4053.

[3] G. Kresse, J. Furthmüller, *Comput. Mater. Sci.* **1996**, *6*, 15.

[4] G. Kresse, J. Hafner, *Phys. Rev. B* **1993**, *47*, 558.

[5] G. Kresse, J. Hafner, *Phys. Rev. B* **1994**, *49*, 14251.

[6] R. Allmann, R. Hinek, *Acta Crystallogr. Sect. A Found. Crystallogr.* **2007**, *63*, 412.

[7] S. Grimme, J. Antony, S. Ehrlich, H. Krieg, *J. Chem. Phys.* **2010**, *132*.

[8] J. Liu, P. Xu, J. Liang, H. Liu, W. Peng, Y. Li, F. Zhang, X. Fan, *Chem. Eng. J.* **2020**, *389*, 124405.

[9] W. Xu, C. Sun, K. Zhao, X. Cheng, S. Rawal, Y. Xu, Y. Wang, *Energy Storage Mater.* **2019**, *16*, 527.

[10] Y. Zhang, J. Wei, M. Jia, Y. Liu, L. Hou, C. Yuan, *ACS Appl. Energy Mater.* **2023**.

[11] M. Imran, N. Akhtar, N. Muzaffar, A. M. Afzal, M. W. Iqbal, S. Safdar, A. A. A. Bahajjaj, S. Mumtaz, M. Z. Ansari, Z. Ahmad, *Phys. Scr.* **2024**, *99*.

[12] H. Sun, H. Liu, Z. Hou, R. Zhou, X. Liu, J. G. Wang, *Chem. Eng. J.* **2020**, *387*, 124204.

[13] M. C. Liu, Y. Xu, Y. X. Hu, Q. Q. Yang, L. Bin Kong, W. W. Liu, W. J. Niu, Y. L. Chueh, *ACS Appl. Mater. Interfaces* **2018**, *10*, 35571.

[14] J. Dai, L. Luo, Z. Tang, Y. Lv, H. Xie, H. Zuo, C. Yang, X. Wang, M. Fan, Y. Xu, L. Dai, *Compos. Sci. Technol.* **2022**, *219*, 109240.

[15] S. Hou, Y. Lian, Z. Xu, D. Wang, C. Ban, J. Zhao, H. Zhang, *Electrochim. Acta* **2020**, *330*, 1.

[16] T. N. Y. Khawula, K. Raju, P. J. Franklyn, I. Sigalas, K. I. Ozoemena, *J. Mater. Chem. A* **2016**, *4*, 6411.

[17] M. Iqbal, N. G. Saykar, A. Arya, I. Banerjee, P. S. Alegaonkar, S. K. Mahapatra, *J. Alloys Compd.* **2021**, *883*, 160705.

[18] P. Pazhamalai, K. Krishnamoorthy, S. Manoharan, S. J. Kim, *J. Alloys Compd.* **2019**, *771*, 803.

[19] Z. Abbas, P. Tiwari, V. Kumar, S. M. Mobin, *Sustain. Energy Fuels* **2022**, *6*, 3872.
